# Liver abscess and septic shock due to *Clostridium perfringens* infection: a case report and literature review

**DOI:** 10.3389/fmed.2025.1575454

**Published:** 2025-04-30

**Authors:** Fangyu Yu, Yuxin Guo, Yujiao Li, Wei Gai, Qianping Zhang, Pochen Li, Ruyi Xu, Lingyao Zhang, Yafeng Zheng, Xiaojing Zhang

**Affiliations:** ^1^Ningbo Municipal Hospital of Traditional Chinese Medicine (TCM), Affiliated Hospital of Zhejiang Chinese Medical University, Ningbo, China; ^2^WillingMed Technology (Beijing) Co., Ltd., Beijing, China

**Keywords:** *Clostridium perfringens*, liver abscesses, metagenomic next generation sequencing, ultrasound-guided drainage, septic shock

## Abstract

*Clostridium perfringens* causes liver abscesses with a low incidence, rapid progression, and high mortality. Within a few days or even within 24 h, patients may progress from a liver abscess to sepsis, multi-organ failure, and potentially death. Diagnosing *Clostridium perfringens* infection by routine microbiological testing (CMT) is often challenging. Here, we present a patient with negative blood cultures who was ultimately diagnosed with a liver abscess due to *Clostridium perfringens* infection, confirmed by metagenomic next-generation sequencing (mNGS). The patient initially presented with fever only, and his blood cultures were negative. Subsequently, the patient’s condition progressed rapidly, and he developed signs of septic shock. Immediately after admission to the ICU, he received combined anti-infective therapy with meropenem and tigecycline, as well as urgent ultrasound-guided puncture and drainage. Blood mNGS identified *Clostridium perfringens* and a variety of anaerobic bacteria, confirming that the pathogen had been covered by empirical antibiotics. Continued anti-infective therapy and drainage improved the patient’s symptoms, and he was eventually discharged from the hospital. Clinicians should be highly suspicious of liver abscesses with negative blood cultures. The use of mNGS to identify the pathogen, appropriate antibiotics, and abscess aspiration and drainage are key to patient survival.

## 1 Introduction

*Clostridium perfringens* is a fast-growing, gram-positive anaerobic bacterium that can tolerate oxygen concentrations of up to 3% and is normally found in the gastrointestinal and reproductive tracts of humans (1–3). Its virulence is primarily determined by more than 20 potent toxins, including α-toxins, β-toxins, and *Clostridium perfringens* enterotoxin (CPE) (4). These potent toxins are released into the bloodstream and cause toxemia through blood circulation, causing rapid deterioration of the patient’s condition, further development of multi-organ failure and even death (5). The clinical manifestations of *Clostridium perfringens* infection range from asymptomatic bacteremia to shock and death (6–8). Liver abscess caused by *Clostridium perfringens* is very rare and has a high mortality rate. The progression from liver abscess to sepsis, multi-organ dysfunction, and even death can occur in just a few days or even hours. Patients with compromised immune systems are more susceptible to *Clostridium perfringens* infection (9). Therefore, early diagnosis and timely intervention are essential for the successful treatment of *Clostridium perfringens* liver abscess and can improve the prognosis of patients. Here, we present a patient with a *Clostridium perfringens* liver abscess. The patient initially presents with fever alone but rapidly progresses to septic shock. The relevant bacteriological cultures were negative, and the diagnosis was confirmed by mNGS. Timely puncture, drainage, and anti-infective treatment resulted in improvement.

## 2 Case presentation

**FIGURE 1:**
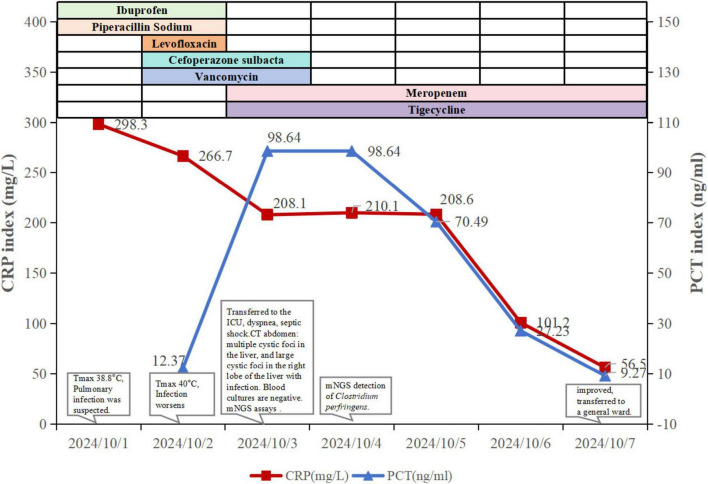
Clinical course of the patient.

**FIGURE 2:**
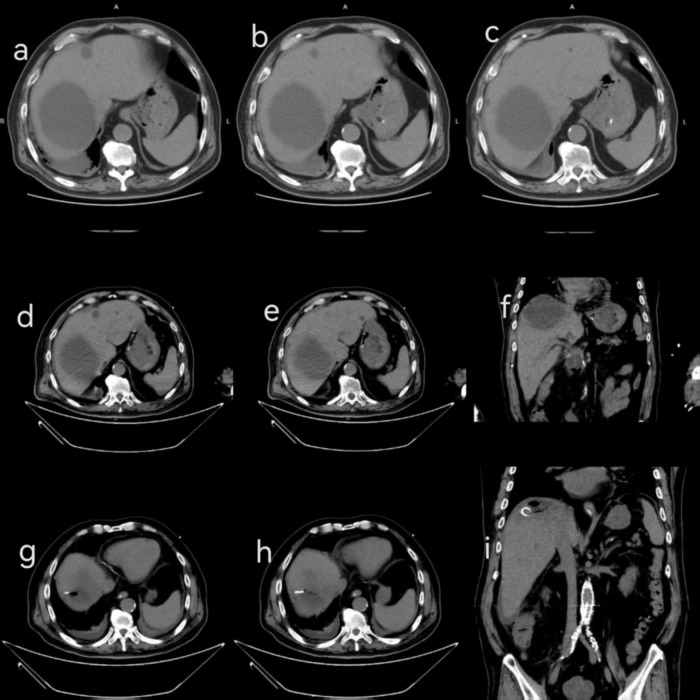
CT findings at different times. **(a–c)** Upon admission, the patient presents with small pleural effusions on both sides, incomplete dilation of the lower lobes of both lungs, miliary lesions, fibrotic lesions, and calcified lesions in both lungs, mild bronchiectasis, and multiple hypodense lesions in the liver. **(d–f)** On October 3, a CT scan revealed postoperative pancreaticoduodenal changes, multiple cystic lesions in the liver, including a large cystic lesion in the right lobe of the liver, and peri-colonic effusion. **(g–i)** On October 4, the right lobe of the liver was punctured and drained, showing multiple cystic lesions in the liver, a small exudate around the ascending colon, mild ascites, and partial absorption of perihepatic fluid compared to before.

A 70-year-old man was admitted to the Department of Endocrinology at Ningbo Hospital of Traditional Chinese Medicine on October 1, 2024, due to recurrent dry mouth and polydipsia for more than 14 years, as well as palpitations for 1 day. He had a history of high blood pressure, diabetes mellitus, tuberculosis, thyroid malignancy, iliac artery dissection, abdominal aortic ulcer, and long-term use of metoprolol succinate extended-release tablets, nifedipine controlled-release tablets, pravastatin, clopidogrel, insulin degludec aspart, acarbose, and metformin. On the day of admission, the patient developed an unprovoked fever, reaching a maximum temperature of 38.8°C, without any other accompanying symptoms (Figure 1). Preliminary laboratory data show elevated white blood cell (WBC) count (9.2 × 10∧9/L) and C-reactive protein (CRP, 298.3 mg/L) levels. Computed tomography (CT) scan of the chest showed a small amount of pleural effusion on both sides with incomplete distension of the lower lobes of both lungs, miliary foci in both lungs, fibrotic foci and calcified foci, mild bronchiectasis, and multiple hypodense foci in the liver (Figures 2a–c). Arterial and venous blood were collected separately for blood cultures. Based on clinical features, the patient was considered to have a pulmonary infection. He received antipyretic and empiric anti-infective therapy with ibuprofen extended-release capsules and piperacillin sodium-tazobactam sodium (4.5 g, q8h). However, in the early hours of October 2, the patient developed a high fever of up to 40°C, accompanied by chills, agitation, and urinary incontinence. He was started on levofloxacin (0.5 g, qd) combined anti-infective therapy and methylprednisolone sodium succinate (40 mg) for anti-inflammatory treatment. After consultations with the ICU, the Departments of Infectious Diseases, Neurology, and Endocrinology, the patient’s anti-infective regimen was adjusted to cefoperazone sulbactam (2 g, q8h) combined with vancomycin (0.5 g, q8h). However, during this period, the patient continued to experience intermittent fever. On 3 October, the patient developed dyspnea and decreased peripheral oxygen (88%), which was considered sepsis and was immediately transferred to the intensive care unit (ICU) for further treatment.

At the time of transfer to the ICU, his vital signs were a body temperature of 39.6°C, heart rate of 140 beats/min, respiratory rate of 37 breaths/min, blood pressure of 158/74 mmHg, and oxygen saturation of 98%. He is conscious, but chills and shivering, with crackles in both lungs, and no other significant findings on physical examination. Laboratory tests show normal WBC (5.7 × 10∧9/L) and total bilirubin (10 μmol/L), with decreased albumin (31.4 g/L) and hemoglobin (124 g/L). Alanine aminotransferase (ALT, 51 U/L, aspartate aminotransferase (AST, 45 U/L), γ-glutamyl transferase (GGT, 115 U/L), neutrophil percentage (NE%, 92.3%), CRP (208.1 mg/L), procalcitonin (PCT, >98.64), interleukin 6 (IL-6, 534.2 ng/ml), and creatinine (131 μmol/L) were significantly elevated. Chest CT showed similar results as before (Figures 2d–f) postoperative pancreaticoduodenal changes, multiple cystic foci in the liver, among which a large cystic foci in the right lobe of the liver with possible infection, pericolonic effusion and effusion. Therefore, the patient was initially diagnosed with septic shock caused by a liver abscess in the ICU. Percutaneous drainage of the liver abscess was performed under emergency ultrasound guidance. Cultures of blood, urine, and sputum were also obtained. The patient received empiric anti-infective therapy with meropenem (1.0 g, q8h) combined with tigecycline (50 mg, q12h) and anti-inflammatory therapy with dexamethasone (5 mg, q12h).

Routine blood and drainage cultures were negative, while urine and sputum cultures detected E. coli, *Enterococcus faecium*, and *Candida albicans*; however, these findings were not entirely consistent with the clinical presentation. To identify the cause, mNGS of the blood was performed on October 3 with the family’s consent. The mNGS analysis followed the protocol used in the previous report (10, 11). DNA and RNA were extracted and prepared into sequencing libraries, and sequencing was performed using the MGISEQ-200 platform (MGI Technology) with a 50 bp single-end sequencing kit. Pathogens were identified based on the specific reads per ten million (RPTM value). On the day following submission (October 4), mNGS detected positive results for *Clostridium perfringens* (1347 RPTM), *Bilophila wadsworthia* (608 RPTM), *Klebsiella pneumoniae* (201 RPTM), *Escherichia coli* (100 RPTM), *Morganella morganii* (79 RPTM), and *Enterococcus casseliflavus* (76 RPTM) (Supplementary Table 1). Based on the mNGS results and clinical symptoms, anaerobic infection due to a liver abscess is strongly suspected, leading to septic shock. Since the pathogens detected by mNGS were already covered by the previous empirical anti-infective therapy, meropenem (1.0 g, q8h) in combination with tigecycline (50 mg, q12h) was continued. Dexamethasone (5 mg, q12h) was used to enhance anti-inflammatory therapy and manage the stress response. Ultrasound-guided drainage of the liver abscess was also performed.

From October 4, the patient’s vital signs improved, and the PCT and IL-6 levels decreased. Abdominal CT showed post-procedure changes in the right lobe of the liver, including multiple cystic foci (Figures 2g–i). The perihepatic effusion was slightly more absorbed than before. After 6 days in the ICU, the patient was stable and transferred to a general ward for further treatment. On October 14, he was discharged from the hospital.

## 3 Discussion and literature review

*Clostridium perfringens* was first discovered in 1891 by William H. Welch during an autopsy (12). *Clostridium perfringens* infections are common in immunocompromised patients and those with post-traumatic or surgical conditions, especially in patients undergoing invasive hepatobiliary and gastrointestinal surgeries, gynecologic procedures, catheter placement, and other invasive procedures. Additionally, advanced age, type II diabetes, and malignancy are risk factors for infection (1). However, there have been reports of patients infected with *Clostridium perfringens* without any high-risk factors, which led to sepsis and death (13, 14).

Bacteria spread via the portal vein or hepatic artery, leading to a liver abscess (3). Liver abscesses are classified based on etiology, with amoebic liver abscess (ALA) and purulent liver abscess (PLA) being the most common types (15). ALA is more prevalent in developing countries and is caused by amoebas (9). PLA is more common in developed countries, typically caused by *Escherichia coli*, *Klebsiella*, and *Streptococcus* (15). In Asia, *Klebsiella pneumoniae* serotypes K1 and K2 are the most common pathogens associated with PLA (16). Cases of PLA caused by *Clostridium perfringens* infection are rare and have an extremely high fatality rate (17). Patients typically present with nonspecific symptoms such as fever, abdominal pain, and/or jaundice in the early stages, which can easily be misdiagnosed as biliary tract disease. Without prompt intervention, gas-producing purulent liver abscess (GPLA) can develop rapidly within days or even hours. It is often complicated by sepsis, massive intravascular hemolysis, renal failure, and eventually MODS, leading to death (3).

We searched PubMed using “*Clostridium perfringens*” and “liver abscesses” and identified 71 cases of *Clostridium perfringens* liver abscess. Of these, 5 cases had incomplete access to the full text due to limitations in publication year or availability, and 66 cases were included in the analysis (Supplementary Table 2). Of the 66 cases reviewed in the literature, the mean age was 68.7 years, with a male-to-female ratio of 42/24. The overall mortality rate was 56.1% (37/66), though the cause of death for 3 patients could not be determined due to unclear descriptions in the literature and patient insistence on discharge. Additionally, 47.0% (31/66) of patients had intravascular hemolysis, and 60.6% (40/66) had sepsis. Among the 37 patients who died, the time of death was not explicitly stated for 2 patients. One patient died of pneumonia after 48 days, and 2 patients died due to progression of underlying cancer (4 weeks and 1 month, respectively). The average survival time for the remaining 33 patients was only 41.9 h (range: 0.8–264 h). Notably, of the 33 patients who died, 69.7% (23/33) received no treatment or only monotherapy (antibiotics or abscess drainage). The average survival time for these patients was just 25.02 h (range: 0.8–264 h). The remaining 10 patients who received two or more treatments, including antibiotics, abscess drainage, and surgery, had a mean survival time of 80.8 h (range: 8–168 h), significantly longer than those who received only one treatment. The Mann-Whitney test indicated a statistically significant difference in survival time between the two groups (*P* = 0.001). This observation underscores the importance of combination therapy, particularly the use of antibiotics and abscess drainage, which can significantly improve patient outcomes and extend survival. Furthermore, univariate binary of variables such as intravascular hemolysis, drainage, and surgery showed that both intravascular hemolysis (OR: 0.184, 95% CI: 0.060–0.570, *P* = 0.003) and drainage (OR: 6.364, 95% CI: 1.979–20.465, *P* = 0.002) significantly influenced mortality. In contrast, two-factor binary logistic regression revealed that intravascular hemolysis (OR: 0.350 95% CI: 0.101–1.280, *P* = 0.114) and drainage (OR: 4.211, 95% CI: 1.204–14.733, *P* = 0.024) continued to show an association with mortality, highlighting that timely abscess drainage is particularly important for reducing patient mortality (Table 1). Among all the included patients, the most common underlying condition was diabetes mellitus (16/66), followed by malignancy (26/66), both of which were more prevalent in older patients. This suggests that immunocompromised populations, particularly older patients with underlying conditions, are more susceptible to *Clostridium perfringens* infection, and clinicians should remain highly vigilant for such infections in these groups.

**TABLE 1:** Univariate and multivariate analysis of survival.

Variable	Univariable analysis		Multivariable analysis	
	**OR (95% CI)**	***P-*value**	**OR (95% CI)**	***P*-value**
Sex	1.467 (0.510, 4.222)	0.477		
Intravascular hemolysis	0.184 (0.060, 0.570)	0.003	0.350 (0.101, 1.280)	0.114
Drainage	6.364 (1.979, 20.465)	0.002	4.211 (1.204, 14.733)	0.024
Surgery	0.938 (0.279, 3.147)	0.917		

In the early stages, accurate diagnosis of patients with *Clostridium perfringens* liver abscess is essential to improve patient survival. First, patients with high-risk factors for *Clostridium perfringens* infection (e.g., cancer, diabetes mellitus) should be vigilant for symptoms such as chills, fever, and abdominal pain. Second, maintain a high index of suspicion for epigastric pain, vomiting, nausea, and impaired consciousness after surgery (particularly for invasive hepatobiliary and gastrointestinal surgeries) (18). Abdominal x-rays, ultrasound, or CT should be performed promptly to confirm or distinguish between GPLA. Once the diagnosis of liver abscess is confirmed, needle aspiration and/or blood cultures should be performed to identify the causative bacteria and initiate appropriate treatment (19).

In our current clinical practice, bacterial culture remains the “gold standard” for microbial diagnosis (20). Bacteriological research has long faced the significant challenge of collecting samples and cultivating them without contamination or exposure to oxygen. Additionally, the *in vivo* growth environment of bacteria differs significantly from that of laboratory culture media. Moreover, it is challenging to replicate the *in vivo* bacterial growth conditions in practice. For these reasons, along with the complexity of the pathogen and the empirical use of antibiotics, the sensitivity of routine bacterial cultures is lower than expected, and the proportion of negative pathogen cultures is higher than what would be expected (21, 22). In this study, the blood samples collected at the time of admission, as well as the blood samples taken 3 days after admission and the drainage culture of the liver abscess, were all negative. Although the admission samples were collected prior to antibiotic administration, the test results were still unsatisfactory. It is likely that the negative culture results after 3 days of admission were due to prior antibiotic exposure. Previous studies have shown that antibiotic exposure reduces the sensitivity of blood cultures, whereas mNGS detection is less affected by prior antibiotic treatment (23, 24). In this context, culture-independent mNGS, which enables unbiased detection of all DNA, is one of the best methods for determining the etiology (25). Although it has limitations, such as generating sequences that match multiple microorganisms and leading to false-negative and false-positive results, as well as its high cost, mNGS remains crucial for etiological diagnosis in cases of negative clinical specimen cultures, antibiotic therapy, or suspected infection with an uncommon pathogen (26).

In cases where there is sufficient clinical evidence to suspect or diagnose a liver abscess caused by *Clostridium perfringens*, early and specific treatment must be initiated to improve patient survival. At present, the most important specific treatment methods include antibiotic therapy, abscess drainage, adjunctive supportive care such as internal environment stabilization, and, if necessary, blood transfusion and blood purification (27). Early use of antibiotics can significantly improve survival (28, 29). The combination of penicillin and clindamycin is considered the most effective treatment for *Clostridium perfringens* infection, as clindamycin inhibits the activity of α-toxins secreted by *Clostridium perfringens* early, greatly reducing the risk of fatal toxemia (30, 31). Multiple studies by Hifumi et al. have also demonstrated that gas gangrene antitoxin can also be used to treat *Clostridium perfringens* sepsis by neutralizing the α toxins of *Clostridium perfringens* (32, 33). Gas gangrene antitoxin is effective in preventing intravascular hemolysis and disease progression. Yamakawa et al. found that antitoxin monoclonal antibodies in combination with gas gangrene antitoxin are a promising treatment for spontaneous *Clostridium perfringens* sepsis (34). The patients in our study maintained treatment with meropenem and tigecycline after obtaining a positive result for *Clostridium perfringens*. Meropenem is a broad-spectrum carbapenem antibiotic that is sensitive to anaerobic bacteria such as *Clostridium perfringens* (35). In addition, prompt drainage is crucial for suspected *Clostridium perfringens* liver abscess. A study by Simon et al. showed that patients who underwent surgical resection and drainage of infected lesions not only had significantly improved survival rates but also a significantly longer survival time compared to those who received conservative management, consistent with our findings (30). These results indicate that puncture drainage, continuous irrigation, and anti-infective therapy immediately after abscess formation are essential for patient survival and prognosis. Hyperbaric oxygen therapy (HBOT) may be a viable adjunctive treatment when abscess drainage or surgical debridement is not feasible, as it reduces toxin production and disrupts the anaerobic environment required for bacterial growth (1). The use of HBOT in acute necrotizing infections may be life saving.

Here, we report a case of liver abscess and septic shock caused by *Clostridium perfringens* infection. The patient’s history of previous pancreaticoduodenal surgery is unknown. Presence of diabetes, hypertension, malignancy, and other underlying diseases. The initial clinical presentation is only high fever and unclear liver abscess on abdominal CT. After antibiotic treatment in the general ward, the disease progressed. Upon transfer to the ICU, a follow-up abdominal CT was performed, and the possibility of a liver abscess was considered. In cases of unknown pathogens, clinicians performed urgent ultrasound-guided puncture and drainage, sent samples for etiological culture, and empirically administered meropenem and tigecycline. However, the patient’s blood culture and drainage fluid culture were negative. To confirm the diagnosis, we performed mNGS testing on the blood, which identified *Clostridium perfringens* infection. This highlights the importance of applying new diagnostic technologies. In the absence of an established etiology, clinicians must identify *Clostridium perfringens* liver abscess early and intervene promptly. *Clostridium perfringens* liver abscess progresses rapidly and has a very high mortality rate.

This study has several limitations. First, all of the 66 cases reviewed were from PubMed and were open access. However, the impact of cases that were unavailable or poorly documented on the study is unpredictable. Second, although all 66 cases reviewed in this study were patients with *Clostridium perfringens* liver abscess, the underlying conditions and specific etiology of the patients varied, and baseline data were not compared across cases. In future research, we need to collect and summarize the etiology to propose better diagnostic and treatment options, to improve the survival rate of patients.

## Data Availability

The original contributions presented in the study are included in the article/Supplementary material, further inquiries can be directed to the corresponding author.
