# Supplementary material for: Liver abscess and septic shock due to Clostridium perfringens infection: a case report and literature review
Source: Front Med (Lausanne). 2025 Apr 30;12:1575454. doi: 10.3389/fmed.2025.1575454 (PMC12074936; doi:10.3389/fmed.2025.1575454)
Supplement: Supplementary file 1 [file Table_1.docx]

Supplementary Material

## Supplementary Tables

**Supplementary Table 1. mNGS results.**

| Sample | Pathogens | RPTM | Relative abundance |
| --- | --- | --- | --- |
| Blood | *Clostridium perfringens* | 1347 | 40.20% |
|  | *Bilophila wadsworthia* | 608 | 18.20% |
|  | *Klebsiella pneumoniae* | 201 | 6.00% |
|  | *Escherichia coli* | 100 | 3.00% |
|  | *Morganella morganii* | 79 | 2.40% |
|  | *Enterococcus casseliflavus* | 76 | 2.30% |

Note: RPTM, reads per ten million.

**Supplementary Table 2. Liver abscess due to Clostridium perfringens has been previously reported.**

| **Title** | **Year** | **Sex** | **Age** | **Underlying diseases** | **Initial symptoms** | **radiographic appearance** | **massive intravascular hemolysis** | **Confirmed method** | **abscess drainage** | **surgury** | **spesis** | **Therapy** | **Outcome** |
| --- | --- | --- | --- | --- | --- | --- | --- | --- | --- | --- | --- | --- | --- |
| A middle-aged lady with a pyogenic liver abscess caused by Clostridium perfringens | 2012 | Female | 50 | rectal cancer with multiple liver secondary | epigastric pain and fever | A bilobed liver abscesses located at right lobe and segment two/three in which the former (15 cm × 12 cm) had central cavitation and the latter (7 cm × 5 cm) had capsular rupture, resulting in loculated fluid and gas collection medial to the stomach | 0 | blood smear | Yes | No | Yes | sulperazone 1 g Q12H and metronidazole 500 mg Q8H intravenously | Death |
| Clostridium perfringens liver abscess complicated by bacteremia | 2015 | Male | 63 | colon cancer | dyspnea, abdominal pain, and fever | A 3.1-cm collection of fluid that contained gas in the left lobe of the liver | 0 | blood cultures | Yes | No | Yes | / | Survival |
| Clostridium perfringens liver abscess after pancreatic resection | 2009 | Female | 65 | pancreatic cancer | spesis | A 9-cm liver abscess | 0 | blood cultures | Yes | No | Yes | piperacillin/tazobactam (4.5 g thrice daily) | Death |
| Intravascular haemolysis and septicaemia due to Clostridium perfringens liver abscess | 2010 | Male | 58 | / | right-sided loin pain and dark coloured urine | a liver abscess and gas collection in the posterior segment of the right hepatic lobe and around the gall bladder | 0 | blood cultures | No | urgent laparotomy | Yes | levofloxacin-benzylpenicillin, clindamycin and metronidazole | Survival |
| Clostridium perfringens sepsis after pancreatoduodenectomy: a case report | 2022 | Male | 70 | T2DM,an ampulla of Vater carcinoma | sudden onset of high-grade fever and chills | an irregular, 20-mm diameter, low-density area in the liver S6 region | 0 | Blood and hepatic abscess cultures | Yes | No | Yes | sulbactam/ampicillin (12.0 g qd) -meropenem (3.0 g qd)-cefmetazole | Survival |
| Gas gangrene caused by clostridium perfringens involving the liver, spleen, and heart in a man 20 years after an orthotopic liver transplant: a case report | 2014 | Male | 71 | orthotopic liver transplant | poorly localized epigastric pain and fever | 2 circular, non–rim-enhancing, totally emphysematous intrahepatic lesions; segment V/VI contained an 8-cm lesion; a smaller 4-cm lesion was located in segment VII | 0 | Blood cultures | No | Yes | Yes | meropenem 1000 mg IV-metronidazole 500 mg, linezolid 600 mg, clindamycin 600 mg, levofloxacin 500 mg,and fluconazole 400 mg IV | Death |
| Microwave ablation of liver metastasis complicated by Clostridium perfringens gas-forming pyogenic liver abscess (GPLA) in a patient with past gastrectomy | 2016 | Male | 84 | gastric adenocarcinoma,total gastrectomy,ultrasound-guided percutaneous MW ablation | abdominal pain and fever | a gas-containing pyogenic liver abscess (GPLA) within segment VIII where the ablation was performed | 0 | hepatic abscess cultures | Yes | No | No | ceftriaxone and metronidazole-tazocin- | Survival |
| Massive haemolysis, gas-forming liver abscess and sepsis due to Clostridium perfringens bacteraemia | 2016 | Male | 65 | T2DM,Chiari type I malformation | epigastric pain, fever, chills and decreased alertness | a lesion in the right lobe measuring 4.5×4.3×4.8 cm | 1 | Blood and hepatic abscess cultures | Yes | No | Yes | ceftriaxone 2 g qd and levofloxacin 500 mg qd-meropenem 1 g q8h and metronidazole 500 mg q8h IV | Survival |
| Liver abscess caused by Clostridium perfringens leading to sepsis | 2024 | Male | 67 | DM | fear of cold | a 59-mm×60-mm×57-mm irregular shape and low-density containing air in liver segment 4, the edges are not clear , which suggested a liver abscess. | 0 | Blood and hepatic abscess cultures | Yes | No | No | cefoperazone-sulbactam | Survival |
| A case of severe intravascular haemolysis | 2022 | Male | 80 | hypertension, diabetes, ischaemic heart disease and Klebsiella liver abscess | right‐sided abdominal pain and symptomatic anaemia | a liver abscess with multiple gas locules with perforation into the intraperitoneal cavity | 1 | hepatic abscess cultures | No | No | Yes | / | Death |
| Liver abscess due to Clostridium perfringens | 1958 | Female | 68 | Diabetes | weakness, anorexia and nausea | a central large round bubble(roentgenogram of the abdomen ) | 0 | Blood and hepatic abscess cultures | No | Yes | Yes | tetracycline IM-streptomycin IM | Death |
| A case of emphysematous hepatitis with spontaneous pneumoperitoneum in a patient with hilar cholangiocarcinoma | 2012 | Female | 80 | hilar cholangiocarcinoma | severe right upper quadrant (RUQ) abdominal pain | hepatic parenchymal emphysema measuring approximately 6.3×4.4 cm in the superior segment of the right liver (S7/S8) with pneumoperitoneum finding in the perihepatic space | 0 | blood cultures | Yes | No | Yes | cefotaxime and metronidazol | Death |
| Clostridium perfringens liver abscess with massive haemolysis | 2010 | Female | 61 | hypertension, diabetes mellitus, and hyperlipidaemia, and had a history of cholecystectomy | fever, upper abdominal pain, and vomiting | the abdomen and pelvis showed the presence of multiple ill-defined hypo-enhancing lesions with air-fluid levels in the left lobe of the liver | 0 | Blood cultures | No | exploratory laparotomy | Yes | cefuroxime (750 mg q8h), ampicillin (1 g every q6h), and metronidazole (500 mg q8h)-meropenem (1 g q12h) and clindamycin (600 mg q12h)-tazobactam-piperacillin (4.5 g q12h) and metronidazole (500 mg every q8h) | Survival |
| Clostridium perfringens Septicemia with Massive Intravascular Hemolysis | 2019 | Female | 77 | pancreatoduodenectomy for cholangiocarcinoma | fever | a hepatic abscess | 1 | Blood cultures | No | No | / | meropenem (2 g/day) and vancomycin (1 g/day) | Death |
| Gaseous liver abscess with Clostridium perfringens sepsis in a patient with neutropaenia | 2019 | Male | 77 | diabetes mellitus, iron overload (idiopathic), atrial fibrillation (on rivaroxaban), myelodysplastic syndrome on chemotherapy | Fever was associated with chills, shortness of breath, and confusion | 2.8 × 1.9 cm2 complex air collection in the right hepatic lobe | 1 | Blood cultures | No | No | Yes | cefepime, vancomycin, and metronidazole | Death |
| Severe sepsis caused by a gas-forming Clostridium perfringens and Klebsiella variicola liver abscess following total pancreatectomy | 2020 | Male | 68 | hypertension and smoking, pancreatic cancer,total pancreatectomy | fever and abdominal pain | a hyperdense area in the sixth liver segment, suspicious of an abscess | 0 | Blood and hepatic abscess cultures | Yes | No | Yes | piperacillin–tazobactam and metronidazole-added meropenem and ciprofloxacin | Survival |
| An Unusual Fatal Outcome of Laparoscopic Cholecystectomy: A Case Report | 2023 | Male | 74 | hypertension, hyperlipidemia, and benign prostatic hyperplasia,laparoscopic cholecystectomy | severe abdominal pain, jaundice, fever, and hematuria | a 5cm gas-filled cavity, located in segment VIII of the liver, near the inferior vena cava | 1 | blood cultures and abdominal fluid | Yes | laparotomy | Yes | piperacillin tazobactam-Meropenem plus Clyndamicin | Death |
| Unexpected Death Associated With Clostridial Sepsis | 2021 | Female | 85 | osteoarthritis, gastroesophageal reflux, postherpetic trigeminal neuralgia, and a mastectomy for carcinoma of the breast | abdominal pain with hypotension, jaundice | / | 1 | blood cultures | No | No | Yes | / | Death |
| Gas-filled Cavities in the Liver | 2018 | Male | 68 | metastatic gastric cancer | chills and shortness of breath | multiple hepatic gas-filled cavities in addition to cancer metastases | 1 | Blood cultures and liver abscess lesions | No | No | Yes | doripenem and azithromycin | Death |
| Multi-Organ Failure Secondary to a Clostridium Perfringens Gaseous Liver Abscess following a Self-Limited Episode of Acute Gastroenteritis | 2015 | Female | 81 | previous cerebrovascular stroke,depression,cholecystectomy and thyroidectomy | lethargy, fever, and deep jaundice | the presence of a 9.9×9.9 cm area of low attenuation in the posterior segment of the right hepatic lobe, containing mostly air | 0 | Blood cultures and liver abscess lesions | Yes | No | Yes | cefepime, metronidazole, and vancomycin-vancomycin and meropenem (IV) and empirical fluconazole IV | Death |
| A patient with sepsis and a gas-forming liver abscess caused by Clostridium perfringens treated with continuous perfusion drainage | 2014 | Male | 64 | / | Fever, diarrhea, disturbance of consciousness | an pneumogenic abscess at S6-7 of the liver | 1 | blood culture | Yes | Yes | Yes | Meloperidine hydrate 1.0g×2/day, Kline Mycin phosphate 600mg×3/day | Survival |
| Gas-Forming Pyogenic Liver Abscess with Septic Shock | 2015 | Male | 77 | hypertension and diabetes mellitus | nonbloody vomiting, nausea, and abdominal pain | an ovoid air and debris containing structure in the left hepatic lobe concerning for hepatic abscess | 0 | liver biopsy | No | No | Yes | vancomycin, piperacillin/tazobactam, and ciprofloxacin IV-metronidazole IV | Death |
| Clostridium perfringens infection after transarterial chemoembolization for large hepatocellular carcinoma | 2015 | Male | 71 | HCC,TACE performed | high fever with chill | a non-uniform abscess filled with gas but no liquid in the right lobe of the liver | 1 | blood culture | No | No | No | piperacillin/tazobactam and levofloxacin | Survival |
| Type A fulminant Clostridium perfringens sepsis indicated RBC/Hb discrepancy; a case report | 2019 | Male | 76 | endoscopic submucosal dissection for early gastric cancer | acute abdominal pain and diarrhea | abscesses with gas in the right posterior lobes of the liver | 1 | blood culture | No | No | Yes | / | Death |
| Multimicrobial sepsis including Clostridium perfringens after chemoembolization of a single liver metastasis from common bile duct cancer | 2000 | Female | 65 | carcinoma of the common bile duct was treated by duodenopancreatectomy, cholecystectomy and hepa ticojejunostomy,TACE performed | fever and mild pain in the right upper abdomen | Subphrenically there was a necrotic region (7 x5 cm) filled with gas and foamy liquid.Small collections of gas were found in the peritoneum, particularly between the liver and the diaphragm close to the abscess. | 1 | blood culture | Yes | No | No | penicillin, 4 x6 million units/day and 3x2 g mezlocillin in combination with 3x 1 g sulbactam- added 240 mg of gentamycin and fluconazole | Survival |
| Massive haemolysis due to sepsis caused by Clostridium perfringens secondary to liver abscess. Presentation of two cases with a similar history | 2018 | Male | 66 | Pancreaticoduodenectomy, adenocarcinoma | Fever, jaundice, general malaise | Hepatic abscess | 1 | / | No | Yes | Yes | / | Death |
|  |  | Male | 63 |  |  |  | 1 |  |  |  |  |  |  |
| Survival in a Case of Emphysematous Cholecystitis With Sepsis Caused by Clostridium perfringens | 2023 | Male | 77 | stroke, type 2 diabetes mellitus, and hypertension | abdominal pain and fever | a gas collection within the gallbladder wall and fluid and gas in the nearby liver tissue | 0 | blood, pus, and bile culture | Yes | No | Yes | cefoperazone/sulbactam and clindamycin-cefmetazole | Survival |
| Fulminant Hepatic Failure and Fatal Cerebral Edema Following Clostridium perfringens Bacteremia: Case Report and Review of Literature | 2017 | Male | 65 | diabetes mellitus and coronary artery disease | abdominal pain | multiple hepatic abscesses containing air fluid levels involving the right hepatic lobe | 0 | blood culture | / | ERCP with sphincterotomy and stent placement | Yes | / | Death |
| Hepatic Clostridium Perfringens Abscess Formation after Radiofrequency Ablation Therapy for Hepatocellular Carcinoma: Report of a Rare Case | 2021 | Female | 63 | chronic hepatitis B-related cirrhosis in Child-Pugh class A status,T2DM,hypertension,HCC,RFAperformed | fever, chills, dyspnea, and abdominal pain | necrotic changes with air component in all 4 treated tumors with favorable abscess formation | 0 | Blood and hepatic abscess cultures | Yes | No | No | / | Survival |
| Clinical and microbiological features of intratumor abscess with bloodstream infection caused by Plesiomonas shigelloides, Citrobacter freundii, Streptococcus mitis/oralis, Clostridium perfringens, and Candida albicans in a patient with cholangiocarcinoma: A case report | 2022 | Male | 77 | hilar cholangiocarcinoma and hypertension,a left and caudate lobectomy, extrahepatic bile duct resection, and biliary reconstruction | fever and abdominal pain | air within the recurrent tumor at the left liver lobectomy resection margin site | 0 | blood cultures,MALDI-TOFMS,16sRNA | No | No | No | ampicillinsulbactam 12 g/day and levofloxacin 500 mg/day-added 100 mg/day micafungin-micafungin was changed to fosfluconazole-oral amoxicillin-clavulanic acid(1,500 mg/day), levofloxacin (500 mg/day), and fluconazole (400 mg/day) | discharged as per his adamant request |
| Multiorgan failure following gastroenteritis: a case report | 2020 | Male | 63 | T2DM,diabetic nephropathy and peripheral vascular disease, ischemic cardiomyopathy, hypertension, and depression | epigastric pain, vomiting, watery diarrhea, generalized muscle aches, and fever | small bowel enteritis, hepatic infarction, portal vein thrombosis suspected to be septic thromboembolism,and several hepatic hypodensities consistent with multiple abscesses, the largest measuring 41mm in diameter | 0 | blood culture | Yes | No | Yes | piperacillin/tazobactam 4.5g IV tid | Death |
| A case of a gas-forming liver abscess caused by Clostridium perfringens after transcatheter arterial chemoembolization | 2018 | Male | 80 | Chronic hepatitis C, hepatocellular carcinoma, hypertension, duodenal papillary carcinoma.pancreaticoduodenectomy,TACE performed | Fever and pain in the right rib | A 6cm gas-producing liver abscess | 0 | Blood and hepatic abscess cultures | Yes | No | No | Meperazone and clindamycin | Survival |
| Sepsis with intravascular hemolysis caused by Clostridium perfringens | 2019 | Female | 67 | / | Fever, general malaise | / | 1 | blood culture | No | No | Yes | / | Death |
| Sudden death caused by Clostridium perfringens sepsis presenting as massive intravascular hemolysis | 2020 | Female | 80 | diabetes mellitus, hypertension, hyperlipidemia, uterine leiomyoma, and lumbar spinal canal stenosis,hysterectomy and unilateral partial hemilaminectomy with bilateral ligamentectomy | vomiting, diarrhea, and pyrexia | multiple gas-filled necrotic cavities in the right lobe of the liver as well as distention of the small intestine | 1 | blood culture | No | No | Yes | 0.5 g of meropenem | Death |
| Case records of the Massachusetts General Hospital. Weekly clinicopathological exercises. Case 42-1989. A 64-year-old woman with a liver abscess, Clostridium perfringens sepsis, progressive sensorimotor neuropathy, and abnormal serum proteins | 1989 | Female | 64 | hyperlipidemia acute myocardial,appendectomy,intermittent hematochezia | high fever | a liver abscess | 0 | blood culture | Yes | No | No | methylprednisolone, 100 mg qd | Survival |
| Gas-forming liver abscess associated with rapid hemolysis in a diabetic patient | 2014 | Male | 65 | T2DM,hypertension and dyslipidemia,coronary stenting | fever, appetite loss, nausea, and upper abdominal pain | a liver abscess 4 cm in diameter with gas formation in the right lobe | 1 | blood culture | No | No | Yes | / | Death |
| Intravascular hemolysis from a Clostridium perfringens liver abscess | 2002 | Male | 80 | DM | afebrile, hemodynamically stable with abdominal pain, severe mental status changes, and jaundice. | a large fluid and gas-filled mass in the liver consistent with an abscess | 1 | blood, abscess and bile cultures | / | / | Yes | / | / |
| Clostridium Perfringens Infection in a Febrile Patient with Severe Hemolytic Anemia | 2016 | Male | 82 | DM | nausea, low-grade fever, and restlessness | an abscess with gas in the left lobe of the liver and emphysematous cholecystitis | 1 | blood culture | No | No | Yes | 0.5 g of meropenem-added 0.6 g of clindamycin | Death |
| A suspected case of Clostridium perfringens sepsis with intravascular hemolysis after transhepatic arterial chemoembolization: a case report | 2019 | Male | 83 | HCC,nonalcoholic steatohepatitis-related cirrhosis,eflux esophagitis, hypertension, and pancreatic carcinoma,pylorus-preserving pancreaticoduodenectomy, TACE performed | fever, nausea, and hematuria | eflux esophagitis, hypertension, and pancreatic carcinoma and he underwent pylorus-preserving pancreaticoduodenectomy approximately | 1 | hepatic abscess cultures(autopsy) | No | No | Yes | piperacillin/tazobactam 4.5 grams and clindamycin 600 mg | Death |
| An Autopsy Case of Fulminant Systemic Infection of Clostridium perfringens With a Diverse Role of Toxins in a Healthy Patient | 2024 | Male | 72 | cholecystitis, glaucoma, and cerebral aneurysm | lower back pain | / | 1 | /(autopsy) | / | / | / | / | Death |
| Massive intravascular haemolysis due to Clostridium perfringens | 2018 | Female | 68 | DM | disturbance and fever | Gas-forming liver abscess | 1 | blood culture (autopsy) | No | No | Yes | vancomycin and piperacillin/tazobactam | Death |
| Subphrenic abscess due to Clostridium perfringens after hepatic resection for hepatocellular carcinoma following emphysematous cholecystitis: Report of a case | 2020 | Male | 69 | chronic hepatitis B,HCC | right subcostal pain and loss of appetite | wall thickening of the gallbladder with air density and an 80-mm tumor with early enhancement in the segment 8 of the liver | 0 | bile and drainage fluid culture | Yes | No | No | Ciprofloxacin and Clindamycin | Survival |
| Sepsis with hemolysis due to a liver abscess in a 60-year-old male patient | 2022 | Male | 60 | Crohn’s disease,biliary resection | Nausea, general weakness, | a 6-centimeter abscess with multiple air envelopes in section 8 of the liver. | 1 | blood culture | Yes | No | No | clindamycin and penicillin G | Survival |
| Clostridial sepsis with massive intravascular hemolysis: rapid diagnosis and successful treatment | 1992 | Male | 61 | DM,myocardial infarction , pulmonary tuberculosis and Whipple's operation of a pancreatic adenocarcinoma | fever with chills | aerobilia with suspected hepatic abscesses | 1 | blood culture | Yes | No | Yes | cefotaxime (2g t.i.d), piperacillin (4g t.i.d) and metronidazole (0.5 g t.i.d)-penicillin G metronidazole and cefotaxime | Survival |
| High Fever After Radiofrequency Ablation of Hepatocellular Carcinoma | 2018 | Male | 70 | hepatitis C infection,myocardial infraction,TACE and RAF performed | high fever with shivering | necrotic lesions concomitant with a substantial amount of air bubbles in the liver | 0 | blood culture | No | No | No | penicillin G and synergic clindamycin | Survival |
| Massive haemolysis because of Clostridium perfringens [corrected] liver abscess in a patient on peritoneal dialysis | 2005 | Male | 65 | diabetes, ischaemic heart disease and renal failure | fever and blood stained peritoneal fluid | fever and blood stained peritoneal fluid | 1 | / | / | / | / | broad-spectrum antibiotics | Death |
| Liver metastasis presenting as pneumoperitoneum | 2005 | Male | 63 | stage III, grade III,Whipple procedure pancreatic adenocarcinoma | sudden-onset abdominal pain associated with vomiting, fever, and chills | pneumoperitoneum | 0 | hepatic abscess cultures | Yes | Yes | No | / | Death |
| A Gas-forming Liver Abscess with Massive Bleeding into the Abscess Cavity Due to a Ruptured Inferior Phrenic Artery | 2021 | Female | 88 | total hip replacement surgery ,T2DMs, hypertension, hyperlipidemia, and hypothyroidism | light epigastric discomfort | a massive abscess 15.5 cm in size that contained an air-fluid level in the right lobe of the liver | 1? | pus culture | Yes | No | No | biapenem-Clindamycin and Metronidazole-levofloxacin | Survival |
| Clostridium liver abscess and massive hemolysis. Unique demise in Fanconi's aplastic anemia | 1984 | Female | 6 | / | cough, chills,and fever | a large cystic mass in the liver with many radiolucent areas | 1 | abscess cultures(autopsy) | No | No | Yes | / | Death |
| Emphysematous Cholecystitis Resulting in Secondary Biliary Cirrhosis: A Rare Complication of Endoscopic Retrograde Cholangiopancreatography | 2013 | Female | 48 | ERCP | chest pain | a large amount of air within and around the gallbladder. | 0 | gallbladder and blood cultures | Yes | laparotomy | No | ampicillin/sulbactam | Survival |
| Hepatic abscess-associated Clostridial bacteraemia presenting with intravascular haemolysis and severe hypertension | 2016 | Male | 58 | / | myalgias and fever | a 5 cm gas-containing liver mass concerning for abscess, with pneumobilia and rupture of the gas into the subcapsular space. | 1 | blood culture | No | No | Yes | piperacillin-tazobactam-added clindamycin | Death |
| Massive intravascular haemolysis during Clostridium perfrigens sepsis of hepatic origin | 2010 | Female | 83 | cholangitis | fever and abdominal pain | a liver abscess | 1 | blood culture | / | / | Yes | / | Death |
| Spontaneous rupture of a Clostridium Perfringens liver abscess into the abdominal cavity | 2009 | Female | 74 | hypertension, embolic right half-life, unexplained kidney failure, urinary septicemia, and Fournier's gangrene | Diarrhea, abdominal pain, fever | a 4 cm long heterogeneous lesion in the third segment of the liver, with multiple heterogeneous nodule genes, rich in free fluid and fluid Discrete expansion of bile in the liver | 0 | Stool and microbiology | No | Yes | Yes | / | Death |
| Spontaneous intrahepatic gas gangrene and fatal septic shock | 2009 | Male | 64 | myelodysplastic syndrome, T2DM,a right hemicolectomy | high fever | a cavitary lesion heterogeneously filled with air | 0 | Blood and hepatic abscess cultures | Yes | No | Yes | Amikacin,piperacilline, tazobactam and voriconazole-vancomycin,piperacilline, tazobactam | Death |
| Tympany over the liver in hepatic abscess caused by Clostridium welchii. Report of a case | 1950 | Male | 67 | pernicious anemia | sever right upper quadrant pain | area of decreased density with fluid level, in a case of abscess of the liver due to Clostridium welchil. | 0 | blood culture | Yes | laparotomy and cholecystectomy | No | Penicillin and streptomycin | Survival |
| A case of freeze-dried gas gangrene antitoxin for the treatment of Clostridium perfringens sepsis | 2015 | Male | 66 | Diabetes mellitus, chronic hepatitis C, diabetic nephropathy, cerebral infarction, subdural hematoma | Fever with general fatigue | A low absorption zone of about 30mm was found in the posterior region of the right lobe of the liver, and air density appeared in the previous low absorption zone 1 hour later | 1 | Blood and hepatic abscess cultures | Yes | No | Yes | Cefmetazole 1g qd- Meropenem 1.5g qd | Death |
| A case of emphysematous hepatitis with spontaneous pneumoperitoneum in a patient with hilar cholangiocarcinoma | 2012 | Female | 80 | hilar cholangiocarcinoma,ndoscopic retrograde biliary stent placed | severe right upper quadrant (RUQ) abdominal pain | ndoscopic retrograde biliary stent was placed | 0 | blood culture | Yes | No | Yes | cefotaxime and metronidazol | Death |
| Severe abdominal pain three weeks after a hemi-hepatectomy | 2015 | Female | 71 | a left-sided hemi-hepatectomy due to liver metastases of a neuroendrocrine tumour of an unknown primary ，ERCP with stent placement. cerebral vascular accident, appendectomy and mild pancreatitis | severe upper  abdominal pain and vomiting | a gaseous configuration in the liver, as seen in infections with gas-forming bacteria such as C. perfringens, | 0 | / | / | / | / | / | / |
| Massive intravascular haemolysis with T-activation and disseminated intravascular coagulation due to clostridial sepsis | 2006 | Male | 80 | cholecystectomy,T2DM | acute onset of rigors, dyspnoea and delirium | a gas-containing liver abscess with pneumobilia | 1 | blood culture | / | / | / | / | Death |
| Solitary pyogenic liver abscess in patients with diabetes mellitus | 1966 | Female | 53 | DM | pain in the right renal angle and hypochondrium,fever and chills | a large cavity separated by 1 cm. of tissue from the lung and extending a long way towards the inferior margin of the liver which, judging from the position of gas in the colon, was not depressed. | 0 | culture | Yes | No | No | ampicillin 500mg q6h-ampicillin 500 mg qd-acycline 500 mg. qd | Survival |
| Intravascular hemolysis due to Clostridium perfringens in an immunocompetent patient | 2009 | Male | 72 | / | Fever, stomach ache | A 2mm diameter cavity containing gas in the liver | 1 | blood culture | / | / | / | imipenem | Death |
| Pneumobilia caused by a clostridial liver abscess: rapid diagnosis by bedside sonography in the intensive care unit | 2007 | Female | 87 | radio frequency ablation of a single metastasis of colonic carcinoma, a right-sided hemicolectomy ,coronary artery disease and anterior myocardial infarction | right upper quadrant abdominal pain febrile, and hypotensive | a gas-containing abscess and pneumobilia and the correct position of the drain | 0 | Blood and hepatic abscess cultures | Yes | No | No | penicillin and clindamycin | Death |
| Case of the season. Emphysematous cholecystitis | 2001 | Female | 55 | DM | pain in the fight upper abdominal quadrant,low-grade fever, and vomiting | air within the lumen and wall of the gallbladder and the pericholecystic region. | 0 | Bile culture | No | cholecystectomy | No | ciprofloxacin and metronidazole | Survival |
| A "fishy" cough: hepatobronchial fistula due to a pyogenic liver abscess | 2015 | male | 49 | chronic recurrent hepatic abscess | coughing persistently. Gross examination of his sputum revealed a ark expectorat | a residual 8cm septated hepatic abscess and a fistulous tract communicating with the bronchial tree | 0 | Cultures of the aspirate | Yes | No | No | ceftriaxone, ciprofloxacin, and metronidazole. | Survival |
| An autopsy case of clostridial liver abscess | 1988 | Male | 68 | DM | Upper abdominal pain, fever, dyspnea | free air just below the right diaphragm | 1 | Liver necrosis tissue culture and blood culture | No | No | Yes | / | Death |
